# Stevens-Johnson Syndrome Following Non-steroidal Anti-inflammatory Drugs: A Real-World Analysis of Post-marketing Surveillance Data

**DOI:** 10.3389/fped.2022.896867

**Published:** 2022-05-06

**Authors:** Qi-hui Shao, Xue-dong Yin, Na Zeng, Zhi-xuan Zhou, Xin-yu Mao, Yan Zhu, Bin Zhao, Zhi-ling Li

**Affiliations:** ^1^Department of Pharmacy, Shanghai Children's Hospital, School of Medicine, Shanghai Jiao Tong University, Shanghai, China; ^2^Shanghai Jiao Tong University School of Medicine, Shanghai, China; ^3^Department of Pharmacy, Peking Union Medical College Hospital, Peking Union Medical College, Chinese Academy of Medical Sciences, Beijing, China

**Keywords:** non-steroidal anti-inflammatory drugs, real-world study, FAERS, spontaneous reporting system, pharmacovigilance, Stevens-Johnson syndrome

## Abstract

**Background:**

The Stevens-Johnson syndrome (SJS) is a severe skin reaction to non-steroidal anti-inflammatory drugs (NSAIDs), and can even be life-threatening. However, there are still few real-world studies to compare the specific differences in the adverse effects of skin and mucosal invasion.

**Methods:**

Disproportionality analysis and Bayesian analysis were devoted to data-mining of the suspected SJS after using NSAIDs based on the FDA's Adverse Event Reporting System (FAERS) from January 2004 to March 2021. The times to onset, fatality, and hospitalization rates of antipyretic analgesic-associated SJS were also investigated.

**Results:**

A total of 1,868 reports of SJS adverse events were identified with NSAIDs. Among 5 NSAIDs monotherapies we studied (acetaminophen, ibuprofen, aspirin, diclofenac and celecoxib), ibuprofen had the highest association with SJS based on the highest reporting odds ratio (ROR = 7.06, 95% two-sided CI = 6.59–7.56), proportional reporting ratio (PRR = 6.98, χ^2^ = 4201.14) and empirical Bayes geometric mean (EBGM = 6.78, 95% one-sided CI = 6.40). However, ibuprofen-associated SJS had the lowest fatality rate (6.87%, *p* < 0.0001) and the highest hospitalization rate (79.27%, *p* < 0.0001). Celecoxib-associated SJS had the latest time to onset (317.56 days, *p* < 0.0001). Diclofenac-associated SJS cases appeared to be associated with the highest risk of death (25.00%, *p* < 0.0001).

**Conclusions:**

The analysis of FAERS data provides a more accurate profile of the incidence and prognosis of SJS after NSAIDs treatment, enabling continued surveillance and timely intervention in patients at risk of SJS following these NSAIDs.

## Introduction

Due to its efficacy in reducing pain and inflammation, non-steroidal anti-inflammatory drugs (NSAIDs) are one of the most popularly used medicines. NSAIDs are traditionally divided into seven groups according to their different chemical structures, the most popular of which are the main derivatives of salicylic acid, acetic acid, enolic acid, anthranilic acid, or propionic acid (1). Meanwhile, NSAIDs can also be classified into non-selective and selective cyclooxygenase (COX) inhibitors according to their mechanism (2). The COX enzyme comes in two forms, COX-1 and COX-2. Non-selective NSAIDs (NS-NSAIDs), represented by aspirin, ibuprofen, acetaminophen, and diclofenac, inhibit COX-1 and COX-2 simultaneously, while selective NSAIDs (S-NSAIDs) specifically inhibit COX-2, represented by celecoxib. However, studies (3, 4) have now found that COX-3 is more sensitive than COX-1 or COX-2 to inhibition by acetaminophen, diclofenac, ibuprofen, and aspirin.

Stevens-Johnson syndrome (SJS) and toxic epidermal necrolysis (TEN) are serious adverse skin drug reactions, mainly involving the skin and mucous membranes. The single difference between them lies in the degree of skin detachment (5). They are rare and quite fatal. The average reported mortality rate of SJS is 1–5%, and that of TEN is 25–35% (6). If the patient is older or has a larger area of skin exfoliation, the mortality rate will be higher. Once SJS develops to TEN, more than 50% of surviving patients suffer from long-term sequelae of the disease (7, 8).

Drugs with higher correlation with SJS were sulfanilamide antibiotics, allopurinol, certain anti-epileptic drugs and nevirapine (9). Since 1957 (10), 30 years after SJS was first reported (11), studies (12–14) of NSAIDs-related SJS have been reported. Nevertheless, most of the evidence for NSAIDs-related SJS comes from case reports and clinical trials. Large sample analysis based on a database is also rare in recent years. Therefore, it is necessary to update our understanding and outline the risks and characteristics of adverse events after NSAIDs treatment for further prevention and management. Hence, we tried to investigate the FDA's Adverse Event Reporting System (FAERS) to evaluate and compare the relationship among acetaminophen, ibuprofen, aspirin, diclofenac, celecoxib, and serious skin adverse events in a large population. Moreover, the differences in onset time, mortality, and hospitalization rate among them were further investigated.

## Methods

### Data Source

A retrospective pharmacovigilance study was conducted using data retrieved from the FAERS database from January 2004 to March 2021.

The FAERS is a public voluntary self-reporting database that provides information on adverse events and medication error reports submitted by a wide range of individuals, including physicians, consumers, and others, from all over the world. Information interchange codes for the FAERS database include demographic and administrative information (DEMO), the Medical Dictionary for Regulatory Activities (MedDRA) preferred terms (PTs) coded for the adverse event (REAC), drug information (DRUG), patient outcomes (OUTC), report sources (RPSR), therapy start dates and end dates for reported drugs (THER), and indications for use (INDI).

### Adverse Event and Drug Identification

Adverse events were investigated by using the MedDRA (Version 24.0) Preferred Terms as follows: epidermal necrosis (10059284), epidermal necrolysis (10014986), Stevens-Johnson syndrome (10042033), toxic epidermal necrolysis (10044223), Stevens-Johnson reaction (10042029). Thus, the MICROMEDEX® (Index Nominum) was used like a dictionary. Acetaminophen, ibuprofen, aspirin, diclofenac, and celecoxib were defined as both brand and generic names in the DRUG file, and the role of the drug was identified as “primary suspected.”

### Data Mining

Based on the basic principles of Bayesian analysis and non-proportional analysis, we applied the reporting odds ratio (ROR), the proportional reporting ratio (PRR), the Bayesian confidence propagation neural network (BCPNN), and the multi-item gamma Poisson shrinker (MGPS) algorithms to investigate the association between NSAIDs and the adverse reactions. The equations and criteria for the four algorithms (15–23) are shown in Table 1. These algorithms were extracted to measure the strength of the association between drugs and adverse events, and if one of the four algorithms met the criteria, it should be considered a positive signal for SJS.

**Table 1:** Summary of major algorithms used for signal detection.

**Algorithms**	Equation*	**Criteria**
ROR	ROR = (a/b)/(c/d)	95% CI > 1, N ≥ 2
	95%CI = e^ln(ROR)±1.96(1/a+1/b+1/c+1/d)∧0.5^	
PRR	PRR = (a/(a+c))/(b/(b+d))	PRR ≥ 2, χ^2^ ≥ 4, N ≥ 3
	χ^2^ = Σ((O-E)^2^/E); (O=a, E = (a+b)(a+c)/(a+b+c+d))	
BCPNN	IC = log_2_a(a+b+c+d)/((a+c)(a+b))	IC025 > 0
	IC025 = e^ln(IC)−1.96(1/a+1/b+1/c+1/d)∧0.5^	
MGPS	EBGM = a(a+b+c+d)/((a+c)(a+b))	EB05 ≥ 2, N > 0
	EB05 = e^ln(EBGM)−1.64(1/a+1/b+1/c+1/d)∧0.5^	

*a: number of reports containing both the suspect drug and the suspect adverse drug reaction. b: number of reports containing the suspect adverse drug reaction with other medications (except the drug of interest). c: number of reports containing the suspect drug with other adverse drug reactions (except the event of interest). d: number of reports containing other medications and other adverse drug reactions. ROR, reporting odds ratio; CI, confidence interval; N, the number of co-occurrences; PRR, proportional reporting ratio; χ^2^, chi-squared; BCPNN, Bayesian confidence propagation neural network; IC, information component; IC025, the lower limit of the 95% two-sided CI of the IC; MGPS, multi-item gamma Poisson shrinker; EBGM, empirical Bayesian geometric mean; EB05, the lower 90% one-sided CI of EBGM.

We calculated the onset time of SJS following acetaminophen, ibuprofen, aspirin, diclofenac, and celecoxib, respectively, which was defined as the interval between adverse event onset date and start date of the administration. Reports with incorrect input or incorrect data input were also excluded. In addition, mortality would be defined as the number of fatal events divided by the total number of NSAIDs-related SJS.

### Statistical Analysis

Descriptive analysis was applied to summarize the clinical characteristics of SJS patients resulting in NSAIDs from the FAERS database. As the data were not normally distributed, non-parametric tests (the Dunn's multiple comparison test for dichotomous variables and the Kruskal-Wallis test when there were more than two subgroups of respondents) were used to compare the time to onset of NSAIDs-associated SJS. Pearson's chi-square test or Fisher's exact test was utilized to compare the mortality and hospitalization rates between different NSAIDs. The statistical significance was set at *p* < 0.001 with 95% confidence intervals. All statistical analyses were performed using GraphPad Prism 8 (GraphPad Software, CA, USA).

## Results

### Disproportionality Analysis and Bayesian Analysis

From January 2004 to March 2021, a total of 1876 cases of SJS-related reports were recorded in the FAERS database. Four hundred and seventy five cases of SJS associated with acetaminophen (APAP) as a suspicious drug, 847 cases associated with ibuprofen, 173 cases associated with aspirin, 202 cases associated with diclofenac, and 179 cases associated with celecoxib were identified. According to the standards of the four algorithms, the severe skin adverse reaction signals were detected for these five NSAIDs, respectively. As shown in Table 2, all of these five drugs had statistically significant ROR, PRR, information component (IC), and empirical Bayesian geometric mean (EBGM). Among them, ibuprofen had the highest association with SJS based on the highest ROR (ROR = 7.06, 95% two-sided CI = 6.59–7.56), PRR (PRR = 6.98, χ*2* = 4201.14) and empirical Bayes geometric mean (EBGM = 6.78, 95% one-sided CI = 6.40). On the contrary, the lowest association with SJS was found in celecoxib.

**Table 2:** Signal detection for NSAIDs-associated Stevens-Johnson syndrome.

**Drugs**	***N***	**ROR**	**PRR**	**IC**	**EBGM**
		**(95% two-sided CI)**	**(**χ^2^)****	**(IC025)**	**(EBGM05)**
APAP	475	4.65 (4.25,5.09)	4.62 (1324.31)	2.19 (2.00)	4.55 (4.22)
Ibuprofen	847	7.06 (6.59,7.56)	6.98 (4201.14)	2.76 (2.58)	6.78 (6.40)
Aspirin	173	2.75 (2.37,3.2)	2.74 (190.68)	1.45 (1.25)	2.73 (2.41)
Diclofenac	202	2.54 (2.21,2.92)	2.53 (186.26)	1.33 (1.16)	2.52 (2.24)
Celecoxib	179	2.54 (2.19,2.94)	2.53 (165.11)	1.33 (1.15)	2.52 (2.23)

*NSAIDs, non-steroidal anti-inflammatory drugs; APAP, acetaminophen; ROR, reporting odds ratio; CI, confidence interval; PRR, proportional reporting ratio; χ^2^, chi-squared; IC, information component; EBGM, empirical Bayesian geometric mean*.

### Descriptive Analysis

The baseline clinical characteristics are summarized in Table 3. Except for the unspecified age, SJS was more likely to occur in young patients (<18 years old) treated with ibuprofen (51.77%) or acetaminophen (36.01%), while aspirin-associated SJS mostly occurred in elderly patients (75–84 years old) (25.63%). In terms of diclofenac and celecoxib, middle-aged patients (45–64 years old) accounted for 34.29 and 47.62% of reported cases respectively, accounting for the majority of the patients in their different age groups. Except for the unspecified data, females made up far more reports than males in the case of all NSAIDs except for acetaminophen, especially celecoxib (68.94 vs. 31.06%), while the proportion of females and males were almost equal in the case of acetaminophen (49.14 vs. 50.86%). As for acetaminophen, ibuprofen, and aspirin, nearly half of the reports were from Europe (43.74, 48.60, and 51.53%, respectively). Diclofenac-associated SJS was mainly reported from Asia (49.43%) and 85.63% of the reports of celecoxib-associated SJS were from North America. Except for celecoxib, other health-professional who were not the pharmacist or the physician accounted for the majority of the reported cases (acetaminophen: 55.82%, ibuprofen: 41.20%, aspirin: 46.54%, diclofenac: 57.23%). For celecoxib, physicians submitted 34.27% of reported cases, which was only lower than the number of cases reported by lawyers (36.36%).

**Table 3:** Clinical characteristics of patients with NSAIDs-associated Stevens-Johnson syndrome collected from the FAERS database (January 2004 to March 2021).

**Characteristics**	**Reports (** ***N*** **, %)**				
	**Acetaminophen**	**Ibuprofen**	**Aspirin**	**Diclofenac**	**Celecoxib**
**Patient age (year)**					
<18	130 (27.37)	337 (39.69)	21 (12.07)	12 (5.94)	0 (0.00)
18–44	120 (25.26)	218 (25.68)	30 (17.24)	52 (25.74)	15 (8.38)
45–64	48 (10.11)	39 (4.59)	31 (17.82)	60 (29.70)	40 (22.35)
65–74	24 (5.05)	19 (2.24)	27 (15.52)	30 (14.85)	12 (6.70)
75–84	34 (7.16)	30 (3.53)	41 (23.56)	10 (4.95)	14 (7.82)
>84	5 (1.05)	8 (0.94)	10 (5.75)	11 (5.45)	3 (1.68)
Unknown	114 (24.00)	198 (23.32)	14 (8.05)	27 (13.37)	95 (53.07)
**Patient gender**					
Female	172 (36.21)	427 (50.29)	102 (58.62)	106 (52.48)	111 (62.01)
Male	178 (37.47)	291 (34.28)	66 (37.93)	86 (42.57)	50 (27.93)
Unknown	125 (26.32)	131 (15.43)	6 (3.45)	10 (4.95)	18 (10.06)
**Area**					
Africa	16 (3.37)	4 (0.47)	7 (4.02)	2 (0.99)	0 (0.00)
Asia	168 (35.37)	70 (8.24)	60 (34.48)	87 (43.07)	11 (6.15)
Europe	199 (41.89)	398 (46.88)	84 (48.28)	61 (30.20)	6 (3.35)
Oceania	2 (0.42)	17 (2.00)	1 (0.57)	0 (0.00)	4 (2.23)
North America	67 (14.11)	308 (36.28)	11 (6.32)	24 (11.88)	137 (76.54)
South America	3 (0.63)	22 (2.59)	0 (0.00)	2 (0.99)	2 (1.12)
Unknown	20 (4.21)	30 (3.53)	11 (6.32)	26 (12.87)	19 (10.61)
**Reporters**					
Consumer	24 (5.05)	113 (13.31)	29 (16.67)	21 (10.40)	21 (11.73)
Lawyer	1 (0.21)	45 (5.30)	0 (0.00)	1 (0.50)	52 (29.05)
Pharmacist	14 (2.95)	39 (4.59)	4 (2.30)	6 (2.97)	4 (2.23)
Physician	147 (30.95)	214 (25.21)	52 (29.89)	40 (19.80)	49 (27.37)
Other health-professional	235 (49.47)	288 (33.92)	74 (42.53)	91 (45.05)	17 (9.50)
Unknown	54 (11.37)	150 (17.67)	15 (8.62)	43 (21.29)	36 (20.11)
**Outcome event**					
Congenital anomaly	0 (0.00)	0 (0.00)	0 (0.00)	0 (0.00)	1 (0.56)
Death	46 (9.73)	58 (6.87)	34 (19.65)	50 (25.00)	21 (11.80)
Disability	17 (3.59)	74 (8.77)	3 (1.73)	7 (3.50)	19 (10.67)
Hospitalization-initial or prolonged	320 (67.65)	669 (79.27)	84 (48.55)	117 (58.50)	84 (47.19)
Life-threatening	92 (19.45)	234 (27.73)	50 (28.9)	47 (23.50)	18 (10.11)
Other serious (Important medical event)	235 (49.68)	420 (49.76)	59 (34.1)	120 (60.00)	147 (82.58)
Required intervention to prevent permanent impairment/Damage	0 (0.00)	16 (1.90)	0 (0.00)	1 (0.50)	5 (2.81)

*NSAIDs, non-steroidal anti-inflammatory drugs; FAERS, FDA Adverse Event Reporting System*.

### Time to Onset of NSAIDs-Associated SJS

**Figure 1:**
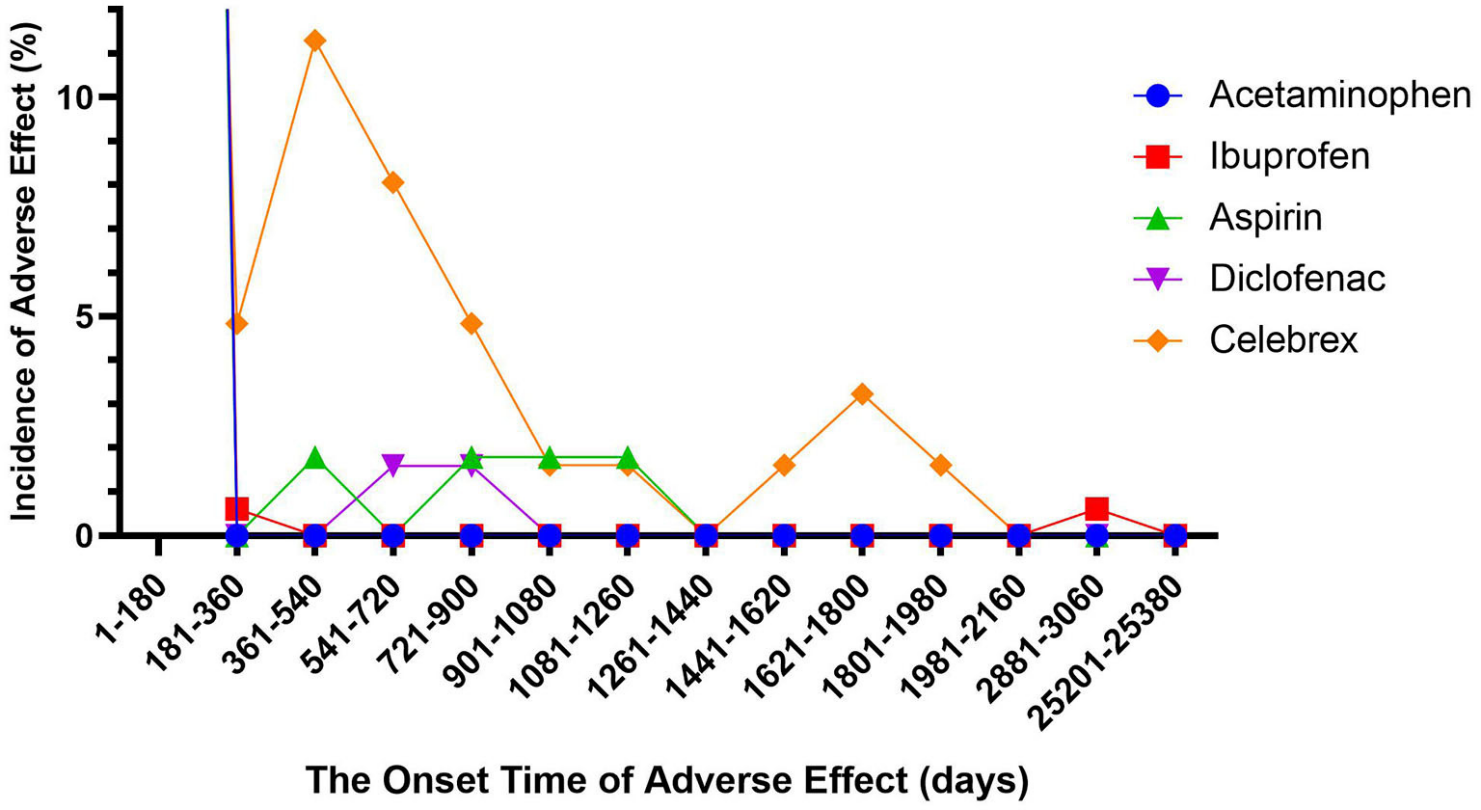
Time to event onset of Stevens-Johnson syndrome following different non-steroidal anti-inflammatory drugs.

We described the time to onsets of SJS for NSAIDs in Figure 1. According to the data, the median onset time of celecoxib-associated SJS was 28 days [interquartile range (IQR) 5.75–490.75], which was significantly longer than that of APAP- (4 days, IQR 1.75–9.75), ibuprofen- (3 days, IQR 1–7) and diclofenac-related SJS (3 days, IQR 0–21) (Dunn's multiple comparison test, *p* < 0.001). Meanwhile, the median onset time of aspirin-associated SJS was 17.5 days (IQR 7.25–28.75), which was also longer than that of the three drugs mentioned above (Dunn's multiple comparison test, *p* < 0.001). However, there was no significant difference between aspirin-related SJS and celecoxib-related SJS. In addition, the average time to onset of celecoxib-associated SJS (317.56 days) and aspirin-associated SJS (81.34 days) was significantly different from that of APAP (7.10 days), ibuprofen (26.33 days) and diclofenac (35.84 days) (Dunn's multiple comparison test, *p* < 0.001).

### Fatality and Hospitalization Due to NSAIDs-Associated SJS

The rate of fatality and hospitalization due to SJS following NSAIDs were assessed to analyze the prognosis of NSAIDs-associated severe skin adverse reactions. The hospitalization rate of ibuprofen-associated SJS was 79.27%, ranking first among the five NSAIDs with absolute advantage (acetaminophen: 67.65%, diclofenac: 58.5%, celecoxib: 47.19%, aspirin: 48.55%) and there were significant differences between ibuprofen and the remaining four NSAIDs (Fisher's exact test, *p* < 0.001). However, the mortality rate of SJS caused by ibuprofen is 6.87%, which was significantly lower than that of aspirin-associated SJS (19.65%) and diclofenac-associated SJS (25.00%) (Fisher's exact test, *p* < 0.001). There was no significant difference between SJS caused by aspirin and diclofenac.

## Discussion

To the best of our knowledge, this study is the largest collection based on the FAERS pharmacovigilance database from January 2004 to March 2021, describing the differences in the vulnerable population, onset time, and adverse outcomes of SJS following NSAIDs (acetaminophen, ibuprofen, aspirin, diclofenac, and celecoxib) in real-world practice. Non-steroidal anti-inflammatory drugs, especially the COX-2 specific inhibitors, were predominantly suspected of triggering SJS (24). However, most previous studies were based on case report series (25, 26), or only focused on a small sample of people in a specific area (27–29). There has been a previous study based on the FAERS database (30), but only some reports of S-NSAIDs in the 2 years after their marketing (up to March 2004) were studied. Therefore, they did not discuss the characteristics of SJS following NSAIDs (both selective and non-selective) from a more comprehensive perspective, nor did they compare SJS following these drugs among these drugs.

Current studies have identified NSAIDs as a suspected cause of SJS (25). Sequelae of Stevens-Johnson syndrome are rare. Reports of SJS caused by these five NSAIDs have only appeared since 2005, and the total number of reports so far is still small. Since there is no specific treatment other than to relieve symptoms, SJS can be life-threatening once it occurs (31). Non-steroidal anti-inflammatory drugs have been widely used for a long time, not only as prescription drugs but also as over-the-counter. Stevens-Johnson syndrome, as one of its serious skin adverse reactions, has a low incidence, but the pain brought by SJS and its sequelae to patients still cannot be ignored.

The mechanism of NSAIDs-associated SJS has not been determined so far. It is hypothesized that liver injury causes toxic retinoid compounds to overflow into the circulation, leading to an endogenous form of hypervitaminosis A and widespread cell apoptosis mediated by granular globulin, eventually manifesting as SJS/TEN (32). Meanwhile, NSAIDs are a leading cause of liver injury across the world (33). Based on the above two points, the reason why NSAIDs lead to SJS is that NSAIDs cause initial damage, which then leads to mitochondrial permeability transformation, and eventually leads to hepatocyte necrosis or apoptosis (34), which finally leads to SJS through the pathways mentioned above. It has also been found that the drug can induce keratinocytes to express the death receptor CD95 (Fas) and its ligand (Fas L), thus significantly increasing the expression of TNF-α, perforin and granzyme B, eventually leading to cell necrosis (35, 36). In addition, the most common drugs related to SJS are the sulfonamide antibiotics (5), so aspirin and celecoxib may be related to SJS due to its sulfonamide moieties.

According to epidemiological results, although all age groups may be affected, the main population still has different priorities. This may be associated with the difference in the population to which they apply. Celecoxib is not recommended for patients younger than 18 years old and therefore there is no data for this age group of patients with celecoxib-associated SJS. Because the World Health Organization (WHO) only recommends acetaminophen and ibuprofen as antipyretic drugs for children, children with fevers are more likely to be exposed to them than other age groups. In addition, because the livers of children are not fully developed and various drug-metabolizing enzymes are not yet complete, they are more likely to develop NSAIDS induced liver damage, which can lead to the progression of SJS. Thus, patients younger than 18 years old are more likely to have SJS. Although aspirin is an antipyretic analgesic drug, it is also often used to reduce the risk of myocardial infarction in patients with cardiovascular risk factors (37). Aging will lead to the decline of liver function in patients (38), which provides an opportunity for the occurrence and development of SJS. At the same time, aspirin might accumulate in the body due to the decreased kidney function in older adults with chronic cardiovascular disease. Thus, aspirin-related SJS are more likely to occur in elder patients.

Based on the FAERS database, the median and average time to onset of severe skin adverse reactions for celecoxib and aspirin were significantly longer than those of APAP, ibuprofen, and diclofenac. However, there is no significant difference between celecoxib and aspirin, or among APAP, ibuprofen, and diclofenac. It may be less convincing to use selective inhibition of COX-2 directly as the cause of the difference in onset. Studies have found that the same patient groups are affected after the use of NS-NSAIDs and S-NSAIDs (25). In addition, aspirin is a non-selective COX inhibitor and celecoxib is a selective COX inhibitor, and the difference between the two does not make a significant difference. However, the median onset time of APAP- and ibuprofen-related SJS, both NSAIDs, is one-fourth to one-fifth that of aspirin-related SJS. From another perspective, we could infer the difference in onset time from the difference in their chemical structure. Aspirin and celecoxib contain sulfonamide moieties whereas the other three drugs do not. The most common drugs that cause SJS are sulfonamide antibiotics, whether it means that sulfa drugs can preserve the potential for serious skin adverse events for a longer period of time. All of this still requires future experiments or longer observations to prove.

According to current research, Stevens-Johnson syndrome usually means an adverse outcome (hospitalization or death). More than almost half of the patients with NSAIDs-associated SJS are likely to be hospitalized, especially ibuprofen (79.27%) and acetaminophen (67.65%). There were significant differences between ibuprofen and the remaining four NSAIDs (Fisher's exact test, *p* < 0.001). Again, this requires special attention in pediatric patients. The young age and short onset time, coupled with the lack of good treatment for SJS itself, may lead to more children being hospitalized. However, the mortality rate of SJS following ibuprofen is 6.87%, which was significantly lower than that of aspirin-associated SJS (19.65%) (Fisher's exact test, *p* < 0.001). This may be because aspirin affects mainly the elderly and the patients it treats are more likely to have cardiovascular disease risk factors and are more likely to die with poorer underlying health conditions. However, it should be noted that SJS itself lacks targeted treatments other than symptom relief, so caution should be exercised regardless of the mortality rate in this study.

Despite the advantages of real-world research and the data mining techniques in this study, inevitably, there are some limitations to this study. First, the signals obtained through Bayesian analysis and disproportionate analysis can only prove that there is a correlation between NSAIDs and SJS, but cannot prove the causal relationship between them. Therefore, the hypothesis generated by the disproportionation analysis needs to be further verified by more reliable methods. Second, FAERS is a spontaneous adverse drug event reporting system, and there may be arbitrariness in reporting. Although we can see the identity of the reporter, the omissions or errors in the report content caused by the recall bias cannot be avoided. For possible duplicate reports (different CASEID but overlapping data), we delete them based on event_dt, age, sex and reporter_country according to the method recommended by the FDA, but important reports may be hidden in the deleted data. Fourth, different drugs are approved at different times, which can lead to more adverse event reports for drugs that have been on the market for long. Take celecoxib and ibuprofen as examples, in the United States, celecoxib was approved for marketing in 2008, while ibuprofen had been on the market as early as 1974. The 34-year difference would bring about a big difference in the number of reports, especially when the start time of the report we selected is January 2004. Fifth, although we can see the statistics of the basic information of many patients, the basic disease status of the patients is not clear, which will bring many confounding factors and bring uncertainty to our analysis. For example, patients may already have underlying chronic conditions such as cardiovascular disease (especially for aspirin), or baseline liver insufficiency, which can affect skin adverse reactions. Although these drawbacks above do exist, the FAERS database can identify signals of NSAIDs and SJS, and further, describe the treatment of these 5 NSAIDs. Our study may provide a new basis for further clinical studies of well-organized NSAIDs-associated SJS.

## Data Availability Statement

The original contributions presented in the study are included in the article/supplementary material, further inquiries can be directed to the corresponding authors.

## Author Contributions

Q-hS analyzed and interpreted data, plotted figures, and wrote the manuscript draft. X-dY, NZ, Z-xZ, and X-yM participated in the interpretation of data. BZ designed and directed the research. Z-lL assisted in preparing this manuscript and providing constructive suggestions. All authors contributed to the article and approved the submitted version.

## Funding

Project sponsored by Innovative Training Program for College Students, School of Medicine, Shanghai Jiaotong University (No. 1521Y424), and Science and Technology Commission of Shanghai Municipality (No. 21DZ2300700).

## Conflict of Interest

The authors declare that the research was conducted in the absence of any commercial or financial relationships that could be construed as a potential conflict of interest.

## Publisher's Note

All claims expressed in this article are solely those of the authors and do not necessarily represent those of their affiliated organizations, or those of the publisher, the editors and the reviewers. Any product that may be evaluated in this article, or claim that may be made by its manufacturer, is not guaranteed or endorsed by the publisher.
